# Effect of Wenxin Granules on Gap Junction and MiR-1 in Rats with Myocardial Infarction

**DOI:** 10.1155/2017/3495021

**Published:** 2017-09-28

**Authors:** Aiming Wu, Mingjing Zhao, Lixia Lou, Jianying Zhai, Dongmei Zhang, Haiyan Zhu, Yonghong Gao, Hongcai Shang, Limin Chai

**Affiliations:** ^1^Key Laboratory of Chinese Internal Medicine of Ministry of Education and Beijing, Dongzhimen Hospital Affiliated to Beijing University of Chinese Medicine, Beijing 100700, China; ^2^National Engineering Research Center for R&D of TCM Multi-Ingredient Drugs, Beijing 100079, China; ^3^Beijing University of Chinese Medicine Institute for Cardiovascular Disease, Dongzhimen Hospital Affiliated to Beijing University of Chinese Medicine, Beijing 100700, China

## Abstract

Myocardial infarction (MI) patients are at high risk of potential lethal arrhythmia. Gap junction and microRNA-1 (miR-1) are both arrhythmia generating conditions. The present study investigated whether Wenxin Granules (Wenxin-Keli, WXKL) could prevent potential lethal arrhythmia by improving gap junctions and miR-1 following MI. Male Sprague-Dawley rats were divided randomly into control, model, metoprolol, low dose WXKL, and high dose WXKL groups. The MI rat model was created by coronary artery ligation. Treatments were administrated intragastrically to the rats for 4 weeks. Conventional transmission electron microscopy was performed to observe the ultrastructure of gap junctions. Quantitative real-time PCR and western blotting were used to detect the expression of miR-1, protein kinase C (PKC), and related proteins. Additionally, a programmatic electrophysiological stimulation test was performed to detect the ventricular fibrillation threshold (VFT). WXKL protected the ultrastructure of the gap junctions and their constituent Cx43 by regulating miR-1 and PKC mediated signal transduction and increased the VFT significantly in the rat MI model. The results suggested that WXKL is an effective alternative medicine to prevent potentially lethal arrhythmia following MI.

## 1. Introduction

Myocardial infarction (MI) remains a predominant cause of mortality and disability worldwide [1]. The prevalence of MI is increasing with the aging global population, leading to serious disease burdens [2, 3]. Reperfusion therapy, such as immediately percutaneous coronary intervention (PCI), has been used frequently in clinical practice and has been remarkably successful in increasing the rate of revascularization and reducing the mortality of MI patients [4]. Although many MI patients survive into the recovery phase which can last years or even decades, patients are still at high risk of sudden cardiac death (SCD) following MI [5]. Ventricular fibrillation (VF) is thought to be a common cause of SCD following MI [6]. Epidemiological data show that in more than half of SCD cases VF occurs as the first symptom [7]. These reports highlight the importance of finding suitable agents to prevent and treat potential lethal arrhythmia following MI.

The recovery phase could be precious for MI patients, because therapeutic preventive measures could decrease the disease progression rate or avoid complications, including arrhythmia. Despite the rapid development of interventional cardiology, drug therapy remains the cornerstone in the prevention and treatment of arrhythmias following MI. Generally, classical antiarrhythmic drugs target mainly the various types of ion channels in the cell membrane. Unfortunately, treating ventricular arrhythmias using an individual family of ion channel antagonist drugs does not reduce mortality in MI patients [8]. The complex etiology of arrhythmia means that it cannot be treated successfully using single target therapy. In addition, the side effects of ion channel antagonist drugs result in them providing a suboptimal solution in terms of the risk/benefit ratio. Although many established therapies are administered to reduce the risk of arrhythmia, MI patients are still confronted with a certain risk of ventricular arrhythmias. Moreover, preventive therapies should be administered over the long term or even lifelong, which sets limits on their acceptable costs.

Fortunately, this problem can be solved partially using alternative medicines, such as traditional Chinese herbal medicine. Recently, traditional Chinese herbal medicine has become more accepted and accessible both in the East and West [9, 10]. Traditional Chinese herbal medicine can provide the first step of early and preventive therapy by reducing the risk of a cardiovascular event [11]. Moreover, many of the traditional Chinese herbal medicines are characterized by good tolerability combined with relatively high efficiency and low price which makes them promising for the long term preventive treatment of members of risk groups. In particular, traditional Chinese medicine has been recognized for its antiarrhythmic potential. Based on the integrative medicine of East and West, the application of traditional Chinese herbal medicine has shown its value in the improvement of existing pathological conditions that have been identified as the mechanisms underlying the generation of arrhythmia. In China, traditional Chinese herbal medicine has been used to prevent and treat cardiovascular system diseases, including arrhythmia, for thousands of years. Several studies have shown that a combination of traditional Chinese herbal medicine and conventional western medicine could prevent significantly the occurrence of malignant arrhythmia and reduce the mortality caused by SCD in MI patients [11–13].

Wenxin Granules (Wenxin-Keli, WXKL) are a traditional Chinese herbal medicine approved by the Chinese state to treat cardiovascular disease [14–19]. Recently, WXKL has been reported to prevent and treat various cardiovascular diseases including cardiac arrhythmias and chronic heart failure [14]. Studies have confirmed that WXKL is a safe and effective alternative medicine that can improve myocardial ischemia, enhance cardiac function, relieve ventricular remodeling, and reduce the occurrence of arrhythmia [15–18]. In addition, an electrophysiological study of WXKL indicated that this agent could produce atrial-selective depression of sodium channel-dependent parameters in canine isolated coronary-perfused preparations via a unique mechanism and is effective in suppressing atrial fibrillation and preventing its induction [19].

As indicated in our previous study [18], WXKL could reverse ventricular remodeling, improve heart function, alleviate histopathological damage, inhibit myocardial apoptosis, and reduce angiotensin II concentrations in rats with MI. Whether WXKL has some additional beneficial effects on potential lethal arrhythmia following MI requires further study. Thus, the present study focused on gap junctions and miR-1, two of the arrhythmia generating conditions, in an attempt to provide additional evidence of WXKL utility to prevent and treat MI.

## 2. Materials and Methods

### 2.1. Animals

Male Sprague-Dawley (SD) rats (180–220 g) were purchased from Beijing Vital River Laboratory Animal Technology Co., Ltd, Beijing, China (license number SCXK (Beijing) 2012-0001). Rats were housed in humidity controlled (60 ± 10)% rooms at (24 ± 1)°C with a 12 h on/12 h off light cycle. The animals were maintained with free access to standard diet and tap water.

### 2.2. Drugs

WXKL was purchased from Shandong Buchang pharmaceutical Co., Ltd, Xi'an, China (SFDA Approval number Z10950026). The main ingredients and quality control of WXKL were the same as those described previously [18]. Metoprolol tartrate tablets were purchased from AstraZeneca Pharmaceutical Co., Ltd, Jiangsu, China (SFDA Approval number H32025391).

### 2.3. Establishment of the MI Rat Model

The MI rat model was established by the coronary artery ligation surgery method used in our previous study [18]. To characterize whether the models were established successfully, echocardiographic examination and routine hematoxylin and eosin (HE) staining were performed as described previously [18].

### 2.4. Design and Allocation

The rats without coronary artery ligation were assigned to control group, and rats with successful coronary artery ligation surgery were divided randomly into the model group, the metoprolol group (12 mg/kg of metoprolol tartrate tablets), the low dose WXKL group (1.35 g/kg of WXKL), and the high dose WXKL group (2.7 g/kg of WXKL), with 13 rats in each group. All drugs were ground and mixed with distilled water before administration. Distilled water was given to the control and model groups. Treatments were administered to the rats intragastrically for 4 weeks, after which the rats were euthanized and dissected to isolate the heart for the subsequent experiments. All rats used in this study received humane care in compliance with the National Institutes of Health Guide for the Care and Use of Laboratory Animals. The study was performed with approval of the Animal Care Committee of Dongzhimen Hospital Affiliated to Beijing University of Chinese Medicine.

### 2.5. Transmission Electron Microscopy (TEM)

Conventional TEM was performed with tissue that was minced into 1-2 mm^3^ cubes and fixed with 2% glutaraldehyde in 0.1 M cacodylate buffer for 1 hour at room temperature (RT). The tissue was postfixed with 2% osmium tetroxide (OsO4) for 1 hour at 4°C and then stained with 2% uranyl acetate for 30 minutes at RT. The tissue was progressively dehydrated using a series of ethanol washes (50, 70, 90, and 100% EtOH) followed by dehydration with propylene oxide and then infiltrated with 1 : 1 propylene oxide : Epon with 1.5% DMP-30 (2,4,6-Tris(dimethylaminomethyl)phenol) for 1 hour. The tissue was then infiltrated with Epon overnight, followed by three more incubations with fresh Epon for 2 hours each. Finally, the tissue was placed in fresh Epon and baked at 60°C for 2 days. The tissue was cut into 70 nm sections using an ultramicrotome, placed on TEM grids, stained with lead citrate, and viewed using a Hitachi 7650 transmission electron microscope operated at 80 kV.

### 2.6. Western Blotting Analysis

All animals were euthanized after 4 weeks of drug administration, and their hearts were immediately harvested and stored in liquid nitrogen until western blotting analyses were performed. The following antibodies were used: Connexin 43 rabbit antibody (Cell Signaling Technology Inc, USA, #3512), anti-Connexin 45/GJA7 antibody - C-terminal (Abcam, ab135474), PKC delta rabbit antibody (Cell Signaling Technology Inc, #2058), p44/42 MAPK (Erk1/2) rabbit antibody (Cell Signaling Technology Inc, #9102), Phospho-p44/42 MAPK (Erk1/2) (Thr202/Tyr204) antibody (Cell Signaling Technology Inc, #9101), Elk-1 rabbit antibody (Cell Signaling Technology Inc, #9182), Phospho-Elk-1 (Ser383) antibody (Cell Signaling Technology Inc, #9181), and SRF (H-300) rabbit antibody (Santa Cruz Biotechnology Inc, USA, sc-13029). The proteins were separated via 10% SDS-PAGE and transferred to nitrocellulose membranes, which were subsequently incubated in primary antibody at 4°C overnight. After the membranes were washed three times with Tris-buffered saline (TBS) and Tween 20 (TBST), they were further incubated in the appropriate horseradish peroxidase-conjugated secondary antibody for 2 hours at RT. Then, ECL visualization was performed. Image J (NIH image, Bethesda, MD, USA) was used to calculate the gray values. The intensity of the target protein was normalized to that of an internal reference to determine the relative expression level of the target protein.

### 2.7. Detection of miR-1

Total RNA was extracted from the myocardial tissue of rats using an RNA extraction kit (Catalog number 217004, Qiagen, Germany) according to the manufacturer's instructions. MicroRNAs were reverse transcribed using a TaqMan® MicroRNA Reverse Transcription Kit (Catalog number 4366596, Applied Biosystems, Foster City, CA, USA) and then used for quantitative real-time PCR using the FastStart Universal SYBR Green Master (Rox) (Catalog number 04913914001, Roche, Switzerland) and 7500 Fast Real-Time PCR System (Applied Biosystems). RNA U6 was used as the internal control. The miR-1 specific primer sequences for quantitative real-time PCR were used for TaqMan MicroRNA Assays (Catalog number 4427975, Applied Biosystem). The expression levels of miR-1 were normalized to U6 and were calculated using the 2^−ΔΔCt^ method [20].

### 2.8. Determination of Ventricular Fibrillation Threshold (VFT)

After 4 weeks of drug administration, rats underwent a second thoracotomy to expose their hearts. A bipolar needle pacing electrode was inserted in the infracted border zone to a depth of about 1 mm. The pacing electrode in the sham-operated group was placed on the corresponding part of the heart. A train of rectangular pulse waves (each train comprised 10 pulse waves, with a pulse duration of 5 ms) was administered at a frequency of 30 Hz. The pacing voltage of the first pulse train started at 1 V and was increased in 1 V increments up to 20 V. The VFT was the minimum voltage that induced ventricular fibrillation.

### 2.9. Statistical Analysis

SPSS software package version 13.0 was used for data analysis. Continuous variables that approximated the normal distribution were expressed as the mean ± standard deviation (SD). Comparisons between groups were subjected to one-way analysis of variance (ANOVA) followed by Fisher's least significant difference (LSD) test. A value of *P* < 0.05 was considered statistically significant.

## 3. Results

### 3.1. The Characteristics of Echocardiography, Electrocardiogram (ECG), and Histology of the MI Rat Model

**Figure 1:**
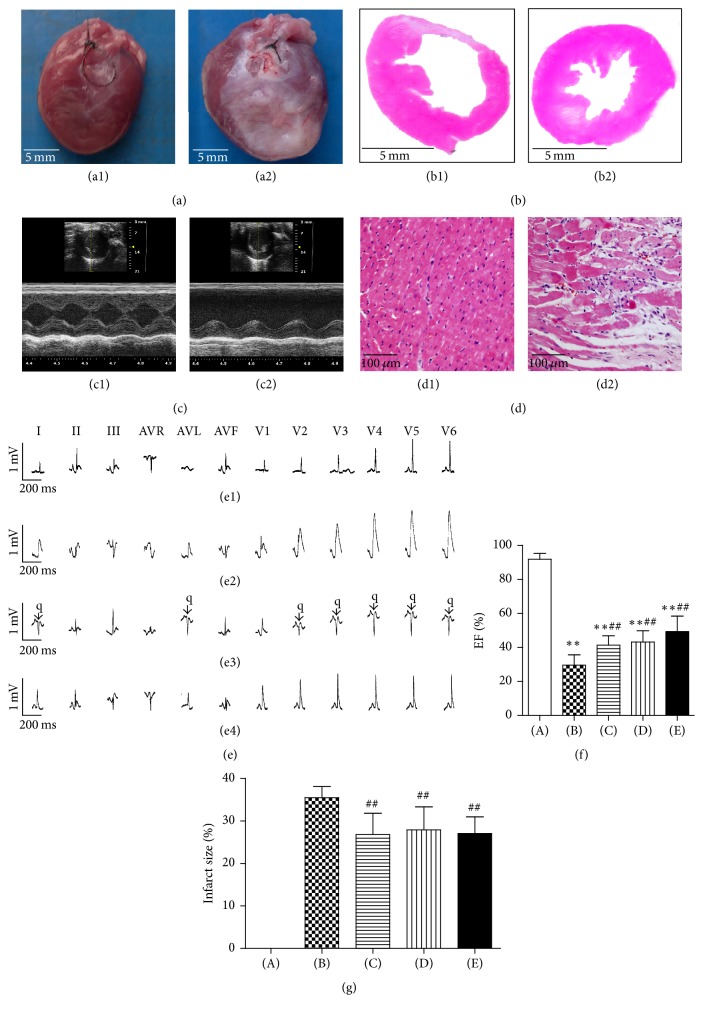
Heart preparation, echocardiography, electrocardiogram (ECG), and pathological sections from normal and MI rats. (a) Preparation of the hearts from the control group (a1) and the model group (a2). (b) Pathological section of the largest cross-section of the control group (b1) and the model group (b2). (c) Typical echocardiography images from the control group (c1) and the model group (c2). (d) Hematoxylin and eosin (HE) staining of myocardial tissue from the control group (d1) and the model group (d2). (e) Typical ECG recordings: the ECG of the model group before coronary artery occlusion surgery (e1); the ECG of the model group 30 minutes after surgery (e2); the ECG of the model group 24 hours after surgery (e3); and the ECG of the control group 24 hours after sham surgery (e4). (f) Comparison of ejection fraction (EF). (g) Comparison of infarct size. (A) Control group, (B) Model group, (C) Metoprolol group, (D) Low dose WXKL group, and (E) High dose WXKL group. Values are expressed as the mean ± SD. ^*∗∗*^*P* < 0.01, versus the control group. ^##^*P* < 0.01, versus the model group.

Four weeks after the coronary artery ligation surgery, cardiac structure and function were measured through electrocardiographic, echocardiographic, and histological examinations. Compared with the control group, the heart in the model was enlarged (Figure 1(a)), the left ventricular chamber was expanded, and the left ventricular wall thickness was reduced (Figure 1(b)). Additional evidence of successful coronary artery ligation was provided from echocardiographic and histological examinations (Figures 1(c) and 1(d)). The ECG recordings of the control and model group rats are shown in Figure 1(e). The ECG showed normal patterns before the coronary artery occlusion surgery (Figure 1(e)1), whereas the ECG of the model group showed a significant increase in ST segments 30 minutes after surgery (Figure 1(e)2), indicative of MI. The ECG of the model group also exhibited pathological Q waves 24 hours after surgery (Figure 1(e)3), as compared to the control group (Figure 1(e)4), which were indicative of MI. As shown in Figure 1(f), the ejection fraction (EF) was lower in the model, metoprolol, low dose WXKL, and high dose WXKL groups compared with that in the control group (all *P* = 0.001). Compared with the model group, the EF was higher in the metoprolol, low dose WXKL, and high dose WXKL groups (*P* = 0.004, *P* = 0.001, and *P* = 0.001, resp.). As shown in Figure 1(g), the infarct size was lower in the metoprolol, low dose WXKL, and high dose WXKL groups compared with that in the model group (*P* = 0.003, *P* = 0.003, and *P* = 0.007, resp.).

### 3.2. Gap Junction Ultrastructure

**Figure 2:**
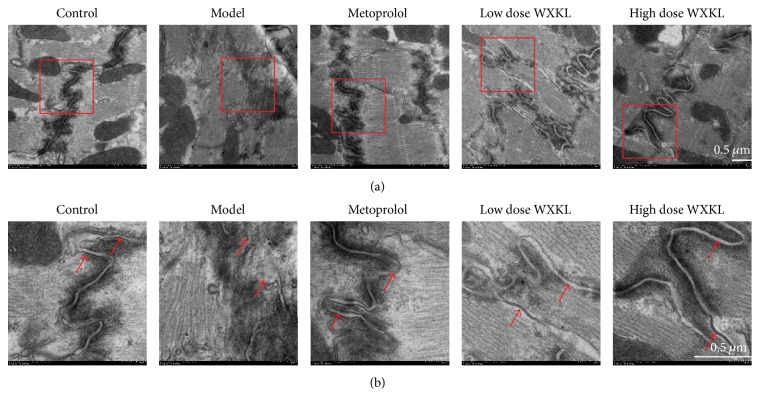
Transmission electron microscopy (TEM) of cardiac tissue sections. Conventional TEM of the intercalated disk is shown in (a) among different groups at 4 weeks after coronary artery occlusion surgery. (b) are magnified views of the red-boxed regions in (a), respectively. Gap junctions are indicated by red arrows. Scale bars = 0.5 *μ*m.

At 4 weeks after coronary artery occlusion surgery, conventional TEM was used to observe the ultrastructure of gap junctions in greater detail (Figure 2). The electron micrographs of normal cardiac tissue demonstrated the location of the gap junctions, which were clearly discernible and continuous within the intercalated disk in the control group. In the model group, gap junctions were blurred, irregular, or discontinuous. Compared with the model group, these ultrastructural changes were alleviated in the metoprolol, low dose WXKL, and high dose WXKL groups.

### 3.3. Expression of Connexin 43 (Cx43) and Connexin 45 (Cx45) Proteins

**Figure 3:**
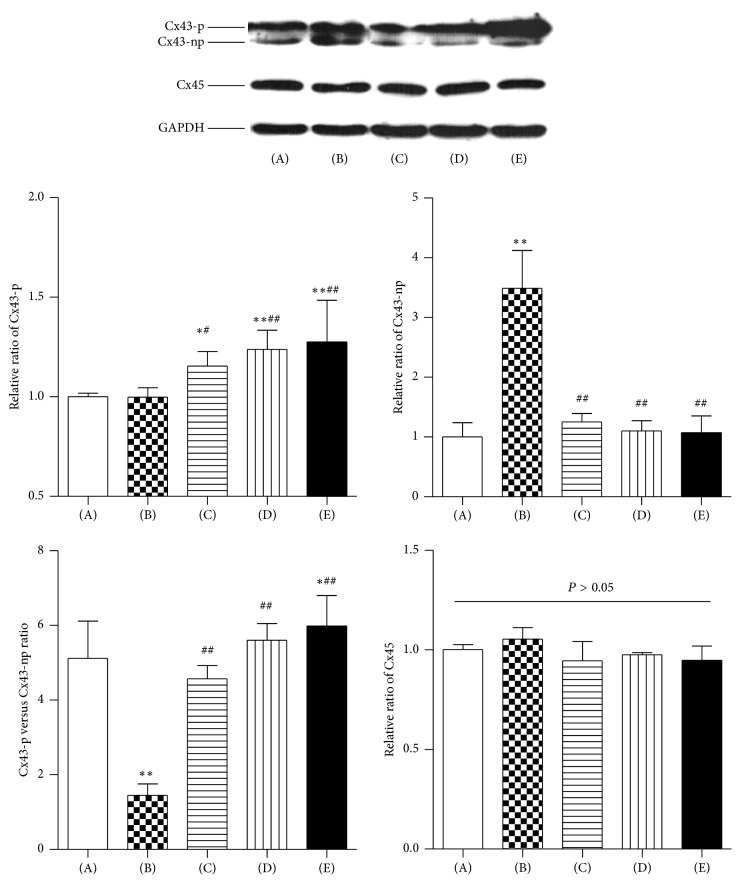
The levels of connexin 43 (Cx43) and connexin 45 (Cx45) proteins. Western blotting analysis was performed to examine the levels of Cx43 and Cx45 in left ventricular tissue among the five experimental groups at 4 weeks after coronary artery occlusion surgery. (A) Control group, (B) Model group, (C) Metoprolol group, (D) Low dose WXKL group, and (E) High dose WXKL group. Values are expressed as the mean ± SD. ^*∗*^*P* < 0.05, ^*∗∗*^*P* < 0.01, versus the control group. ^#^*P* < 0.05, ^##^*P* < 0.01, versus the model group.

Western blotting analysis was performed to examine the levels of Cx43 and Cx45 in left ventricular tissue among the five experimental groups. As shown in Figure 3, there was no significant difference in phosphorylated Cx43 (Cx43-p) levels between the control group and the model group (*P* = 0.937). The level of Cx43-p was higher in the metoprolol, low dose WXKL, and high dose WXKL groups compared with that in the control group (*P* = 0.038, *P* = 0.002, and *P* = 0.001, resp.). Compared with the model group, a similar pattern of Cx43-p was observed in the metoprolol, low dose WXKL, and high dose WXKL groups (*P* = 0.032, *P* = 0.002, and *P* = 0.001, resp.). The level of nonphosphorylated Cx43 (Cx43-np) increased significantly in the model group compared with the control group (*P* = 0.001). Compared with the model group, the level of Cx43-np was lower in the metoprolol, low dose WXKL, and high dose WXKL groups (all *P* = 0.001). The Cx43-p/Cx43-np ratio decreased significantly in the model group compared with that for the control group (*P* = 0.001). Compared with the model group, the Cx43-p/Cx43-np ratio was higher in the metoprolol, low dose WXKL, and high dose WXKL groups (all *P* = 0.001). In terms of the expression of Cx45, there were no statistically significant differences among the five experimental groups (*P* > 0.05).

### 3.4. Relative Expression of miR-1

**Figure 4:**
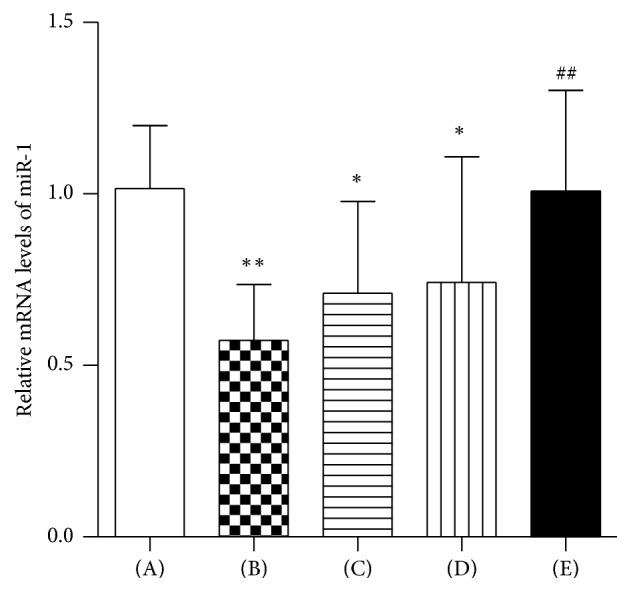
Relative Expression of miR-1. Quantitative real-time PCR was performed to examine the relative expression of miR-1 in left ventricular tissue among the five experimental groups at 4 weeks after coronary artery occlusion surgery. (A) Control group, (B) Model group, (C) Metoprolol group, (D) Low dose WXKL group, and (E) High dose WXKL group. Values are expressed as the mean ± SD. ^*∗*^*P* < 0.05, ^*∗∗*^*P* < 0.01, versus the control group. ^##^*P* < 0.01, versus the model group.

Quantitative real-time PCR was performed to examine the relative expression of miR-1 in left ventricular tissue among the five experimental groups. The relative expression of miR-1 was normalized against that of the U6 endogenous control. Compared with the control group, the relative expression of miR-1 decreased in the model, metoprolol, and low dose WXKL groups (*P* = 0.002, *P* = 0.032, and *P* = 0.046, resp.). Compared with the model group, the relative expression of miR-1 increased in the high dose WXKL group (*P* = 0.005) (Figures 4 and S1 in Supplementary Material available online at https://doi.org/10.1155/2017/3495021).

### 3.5. The Levels of Protein Kinase C (PKC) and Related Proteins

**Figure 5:**
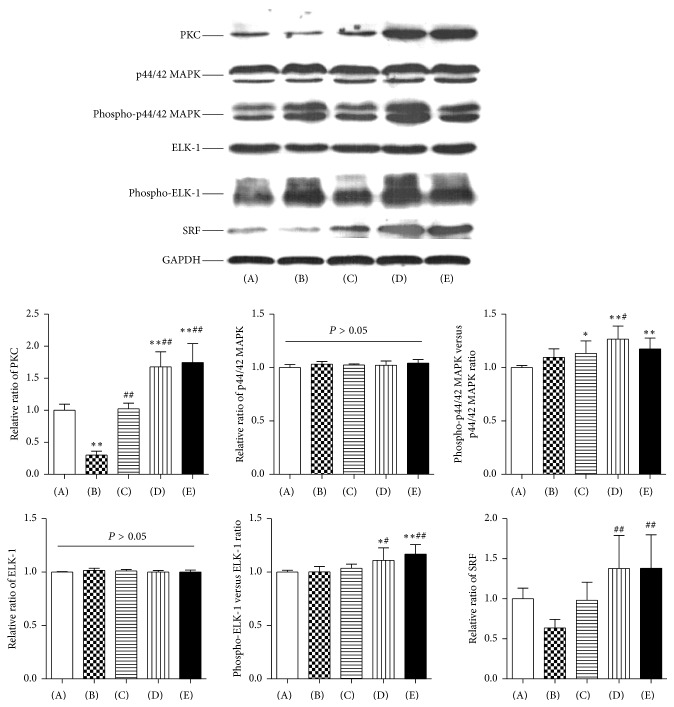
The abundances of PKC and related proteins. Western blotting analysis was performed to examine the abundance of PKC and related proteins in left ventricular tissue among the five experimental groups at 4 weeks after coronary artery occlusion surgery. (A) Control group, (B) Model group, (C) Metoprolol group, (D) Low dose WXKL group, and (E) High dose WXKL group. Values are expressed as the mean ± SD. ^*∗*^*P* < 0.05, ^*∗∗*^*P* < 0.01, versus the control group. ^#^*P* < 0.05, ^##^*P* < 0.01, versus the model group.

Western blotting analysis was performed to examine the levels of PKC and related proteins in left ventricular tissue among the five experimental groups (Figure 5). Compared with the control group, the level of PKC decreased in the model group (*P* = 0.001) and increased in the low dose WXKL and high dose WXKL groups (both *P* = 0.001). Compared with the model group, the expression of PKC increased in the metoprolol, low dose WXKL, and high dose WXKL groups (all *P* = 0.001). For p44/42 MAPK and ELK-1, there were no statistically significant differences among the five experimental groups (*P* > 0.05). Compared with the control group, the expression of phospho-p44/42 MAPK increased in the metoprolol, low dose WXKL, and high dose WXKL groups (*P* = 0.041, *P* = 0.001, and *P* = 0.009, resp.), while no significant differences were observed between the control and model groups (*P* = 0.129). The level of phospho-p44/42 MAPK increased in the low dose WXKL group compared with that in the model group (*P* = 0.010). The expression of phospho-ELK-1 increased in the low dose WXKL and high dose WXKL groups compared with the control group (*P* = 0.026 and *P* = 0.001, resp.). Compared with the model group, similar levels of phospho-ELK-1 were observed in the low dose WXKL and high dose WXKL groups (*P* = 0.028 and *P* = 0.002, resp.). In our analysis of the levels of serum response factor (SRF) in the five experimental groups, the level of SRF increased in the low dose WXKL and high dose WXKL groups compared with that in the model group (both *P* = 0.001); however, the differences were not significant compared with that in the control group (*P* = 0.054 and *P* = 0.052, resp.).

### 3.6. Ventricular Fibrillation Threshold (VFT)* In Vivo*

**Figure 6:**
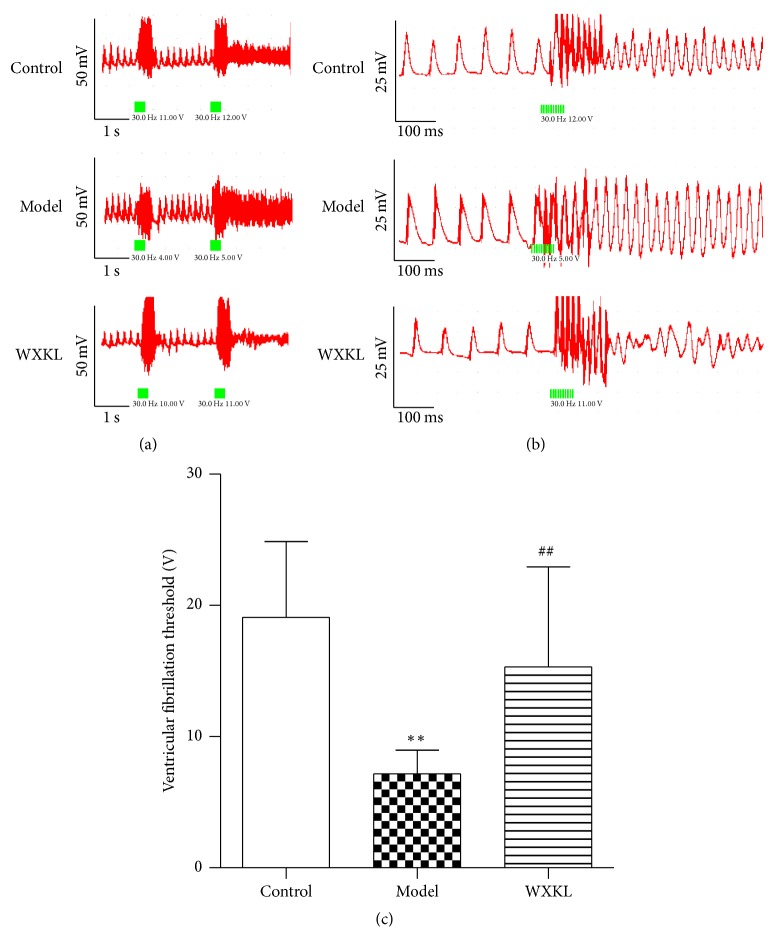
Ventricular fibrillation threshold (VFT) measurements. To confirm the beneficial effect of WXKL for potential lethal arrhythmia following MI, programmatic electrophysiological stimulation was performed after 4 weeks of MI* in vivo*. (a) The compressed waveform of the epicardial electrogram recordings. (b) The expanded waveform of the epicardial electrogram recordings. (c) Comparison of VFTs. Values are expressed as the mean ± SD. ^*∗∗*^*P* < 0.01, versus the control group. ^##^*P* < 0.01, versus the model group.

To confirm the beneficial effect of WXKL for potential lethal arrhythmia following MI, a programmatic electrophysiological stimulation test was performed after 4 weeks of MI. The rats in the WXKL group were given oral doses of 1.35 g/kg of WXKL per day for 4 weeks. As shown in Figure 6(a), the compressed waveform of the epicardial electrogram recordings changed dramatically after the second train of electrical stimulation pulses (indicated in green), compared with the previous one.  Figure 6(b) shows detailed tracings for VF through the expanded waveform of the epicardial electrogram recordings. As shown in Figure 6(c), the VFT decreased significantly in the model group compared with that in the control group (*P* = 0.001). Compared with the model group, the VFT increased significantly in the WXKL group (*P* = 0.007).

## 4. Discussion

We could draw the following conclusions from the present study. (1) WXKL treatment protected the ultrastructure of gap junctions within the intercalated disk after MI. (2) WXKL treatment alleviated the abnormal levels of Cx43 phosphorylation significantly following MI. (3) WXKL treatment increased the expression of miR-1, PKC, phospho-p44/42 MAPK, phospho-Elk-1, and SRF significantly. (4) WXKL treatment increased the VFT significantly.

The existing cardiac pathological conditions are the substrates of potential lethal arrhythmia following MI. In addition to various ion channels in the cell membrane, abnormal gap junctions are increasingly being recognized as critical factors in susceptibility to post-MI arrhythmias [21]. Gap junctions are clusters of transmembrane channels that mediate the electrical coupling of adjacent cells and allow cell-to-cell transfer of molecules of <1 kDa in molecular weight, including ions and small molecules. In cardiac tissue gap junctions are formed primarily at the intercalated disk, which is the site of contact between the ends of cardiomyocytes. This arrangement accounts for anisotropic current flow, with conduction progressing rapidly in the direction of tissue fiber orientation [22]. In the present study, we demonstrated that the ultrastructure of gap junctions within the intercalated disk was severely disrupted at 4 weeks after MI; that is, they looked blurred, irregular, or discontinuous under conventional TEM. Arrhythmia generating conditions induced by persistent ischemia following MI modify the intercellular coupling by altering the conductance of gap junctions in the normal electric propagation pathway of the heart [23]. Pathological changes to gap junctions modify conduction and have been implicated causally in reentrant arrhythmogenesis [24]. As we learn more about gap junctions and their constituent connexins in the heart, it appears that they may also be a potential therapeutic target. Some studies have shown that therapy to improve gap junctions can increase ventricular conduction velocity, delay the onset of spatially discordant alternans, and decrease the susceptibility to arrhythmia [25, 26]. In the present study, both low dose and high dose WXKL could protect the ultrastructure of gap junctions to a certain extent. This might represent an important mechanism by which WXKL is able to inhibit arrhythmia.

Gap junction channels are assembled from two hexameric hemichannels, or connexons, (formed by connexins) which dock together at contacts between cells [22]. Cx43 is the primary gap junction channel protein that is highly expressed within mammalian ventricular muscle at the intercalated disk. Cx43 phosphorylation is implicated widely in gap junction function. Abnormal levels of Cx43 phosphorylation appear to be a critical factor in disrupting cardiac rhythms. In addition to Cx43, another gap junction protein, Cx45, although present at relatively low levels in the working myocardium, is absolutely required to maintain cardiac rhythms [27]. In the present study, we demonstrated that both doses of WXKL increased the expression of Cx43-p significantly, decreased the expression of Cx43-np, and, especially, increased the Cx43-p/Cx43-np ratio. However, no significant effect of WXKL was found on Cx45 in present study. Phosphorylation events are essential for the correct composition and function of complete dodecameric gap junction channels. Cx43 is phosphorylated extensively on its carboxy terminus, which regulates the trafficking, assembly, and permeability of channels. The phosphorylated form appears after Cx43 has reached the plasma membrane and formed gap junctions; thus, Cx43-p is the majority form of Cx43 [28, 29]. A decreased Cx43-p/Cx43-np ratio might induce a proarrhythmic state under pathological conditions. Maintaining the initial ratio of Cx43-p relative to Cx43-np might be an additional beneficial effect of WXKL on gap junctions in the rat model of MI. Although Cx45 also affects arrhythmic susceptibility, unfortunately the present results did not reveal an intervention effect of WXKL on Cx45.

Gap junctions and their constituent connexins are tightly regulated in response to changes in voltage, calcium concentration, redox reactions, phosphorylation, protein interactions, and changes in pH [30]. Among these, phosphorylation events are essential for connexins [30]. Additionally, a well-documented control mechanism of connexins synthesis is through the action of small single-stranded RNAs called microRNAs (miRNAs or miRs) [31]. To reveal the specific regulation mechanism of WXKL in improving Cx43 expression and phosphorylation, we further detected the expression of miR-1, PKC, and related proteins. The results showed that WXKL increased the expressions of miR-1, PKC, phospho-p44/42 MAPK, phospho-ELK-1, and SRF significantly. miRNAs are small (approximately 22 nucleotides) noncoding RNAs that regulate gene expression posttranscriptionally by binding complementary sequences within mRNA transcripts [32]. The human genome encodes over 1800 miRNAs, which target about 60% of human genes [33]. Consequently, miRNAs are likely to regulate many complex processes in the body. Indeed aberrant expression of various miRNAs has been implicated in numerous disease states [34]. They also participate in the pathogenesis of cardiovascular disease, including atherosclerosis, coronary artery disease, myocardial infarction, heart failure, and cardiac arrhythmias [35–37]. miR-1 is one of the most abundant miRNAs in the heart and has been reported to regulate cardiac electrical remodeling and structural remodeling, which are identified mechanisms underlying the generation of arrhythmia [38]. miR-1 is absolutely required to maintain cardiac rhythms. Evidence supporting a role for miR-1 in arrhythmogenesis came from a mouse model lacking miR-1-2; these mice had a spectrum of abnormalities, including ventricular septal defects in a subset that suffered early lethality, and cardiac rhythm disturbances in those that survived [39]. Additionally, inhibiting miR-1 expression might induce cardiac hypertrophy and arrhythmia [40]. However, the effect of miR-1 on arrhythmogenesis is a matter of debate. miR-1 is involved in the occurrence of arrhythmia via the posttranscriptional regulation of connexin expression [41]. When miR-1 was overexpressed in normal and infarcted rat hearts, conduction slowed and susceptibility to arrhythmia increased [41]. In the present study, we demonstrated that the relative expression of miR-1 decreased in the ischemic heart after 4 weeks of MI. Thus, our finding was in line with that of previous studies. Similar results were also observed in clinical studies. miR-1 was downregulated in autopsy samples of infarcted heart tissue from patients with MI [42]. miR-1 was also downregulated in patients with symptomatic heart failure and its expression decreased according to the severity of New York Heart Association (NYHA) class [43]. miR-1 targets* GJA1*, which encodes Cx43 [44]. This explains why the expression of Cx43-np was significantly increased in the MI rats. However, there was no reciprocal increase in Cx43-p because of downregulation of PKC in the MI rats. PKC is a serine/threonine kinase that was described as a Ca- and phospholipid-dependent protein kinase. Kinases are protein switches that activate other proteins by adding a phosphate group to them via phosphorylation. On the one hand, PKC directly mediates the phosphorylation of Cx43 [45]. On the other hand, PKC led to phosphorylation and translocation of p44/42 MAPK, also known as extracellular signal-regulated kinase 1/2 (ERK1/2), and then activated a transcription factor, ELK-1 [46]. Phospho-ELK-1 can form a ternary complex with the serum response elements (SRE) and SRF, thereby activating SRF [47]. SRF, a cardiac enriched transcription factor, might regulate the expression of miR-1 directly [48]. As indicated in present study, WXKL significantly increased the expression of miR-1, PKC, phospho-p44/42 MAPK, phospho-ELK-1, and SRF. These findings provide one possible mechanism by which WXKL protects Cx43 and that this protection is dependent on miR-1 and PKC mediated signal transduction.

The results presented in this study indicate that WXKL might be a suitable and effective alternative medicine to prevent potentially lethal arrhythmia following MI. To prove our hypothesis, we performed an additional programmatic electrophysiological stimulation test after 4 weeks of MI* in vivo*. The results supported our conclusions: WXKL treatment could significantly increase the VFT in rats with MI.

The observed beneficial effects of WXKL can be attributed to its main ingredients, including* Panax notoginseng*,* Codonopsis pilosula*, and* Nardostachyos Radix et Rhizoma*. Modern pharmacological studies show that the active ingredients of* Codonopsis pilosula* play a very important role in treatment of cardiovascular system diseases and can attenuate calcium influx and apoptosis induced by angiotensin II plus Leu (27) insulin-like growth factor II in H9c2 cardiomyoblasts [49]. Several studies have reported that* Panax notoginseng* has many cardiovascular protective effects, including proangiogenesis, antiapoptosis, decreasing oxidative stress, repressing inflammatory cascade, and endothelium-dependent vasodilation effects [50–52]. The volatile oil of* Nardostachyos Radix et Rhizoma* can inhibit oxidative stress-induced cell death via reactive oxygen species scavenging and Akt activation in H9c2 cardiomyocyte [53]. These ingredients are the material basis for the cardioprotective effects of WXKL.

## 5. Conclusions

The present study provided direct evidence that WXKL could protect the ultrastructure of gap junctions and their constituent Cx43 by regulating miR-1 and PKC mediated signal transduction, while also significantly increasing the VFT in a rat MI model. These findings suggested that WXKL might be a suitable and effective alternative medicine to prevent potentially lethal arrhythmias following MI.

## Supplementary Material

The standard curve, the amplification plot, and the melt curve plot of miR-1.
