# Supplementary material for: Effect of Wenxin Granules on Gap Junction and MiR-1 in Rats with Myocardial Infarction
Source: Biomed Res Int. 2017 Sep 28;2017:3495021. doi: 10.1155/2017/3495021 (PMC5637836; doi:10.1155/2017/3495021)
Supplement: Supplementary file 1 — The standard curve, the amplification plot, and the melt curve plot of miR-1. [file 3495021.f1.pdf]

Supplementary material

Relative Expression of miR-1

The amplification efficiency was calculated by standard curve. As shown in Figure S1 (a), the amplification efficiency of miR-1 was 102.638%. The amplification plot of miR-1 showed "s" shape which indicated the normal amplification (Figure S1 (b)). At the end of the PCR cycles, melting curve analysis was performed to validate the specific generation of the expected PCR product (Figure S1 (c)).

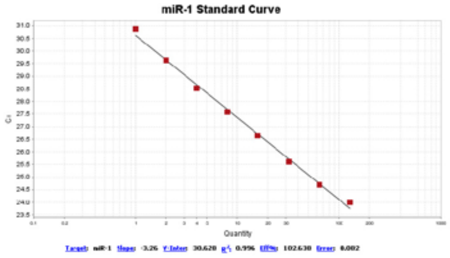

(a)

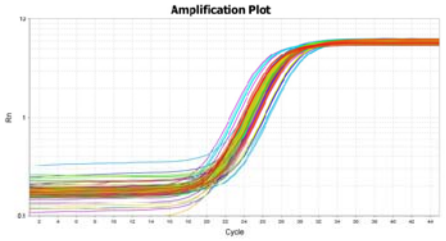

(b)

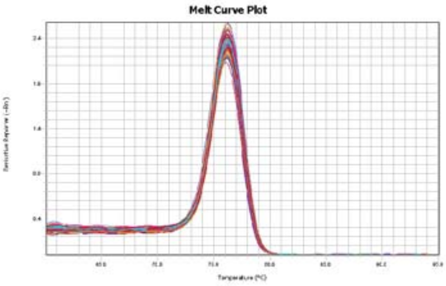

(c)

Figure S1: Relative Expression of miR-1. Quantitative real-time PCR was performed to examine the relative expression of miR-1 in left ventricular tissue at 4 weeks after coronary artery occlusion surgery. (a) The standard curve of miR-1. (b) The amplification plot of miR-1. (c) The melt curve plot of miR-1.
